# *Candidatu*s Alkanophaga archaea from Guaymas Basin hydrothermal vent sediment oxidize petroleum alkanes

**DOI:** 10.1038/s41564-023-01400-3

**Published:** 2023-06-01

**Authors:** Hanna Zehnle, Rafael Laso-Pérez, Julius Lipp, Dietmar Riedel, David Benito Merino, Andreas Teske, Gunter Wegener

**Affiliations:** 1 Max Planck Institute for Marine Microbiology, Bremen, Germany; 2 MARUM, Center for Marine Environmental Sciences, University of Bremen, Bremen, Germany; 3 Faculty of Geosciences, University of Bremen, Bremen, Germany; 4 Systems Biology Department, Centro Nacional de Biotecnología (CNB-CSIC), Madrid, Spain; 5 Max Planck Institute for Multidisciplinary Sciences, Göttingen, Germany; 6 Department of Earth, Marine and Environmental Sciences, University of North Carolina at Chapel Hill, Chapel Hill, NC USA; 7 Present Address: Biogeochemistry and Microbial Ecology Department, Museo Nacional de Ciencias Naturales (MNCN-CSIC), Madrid, Spain

**Keywords:** Biogeochemistry, Archaea

## Abstract

Methanogenic and methanotrophic archaea produce and consume the greenhouse gas methane, respectively, using the reversible enzyme methyl-coenzyme M reductase (Mcr). Recently, Mcr variants that can activate multicarbon alkanes have been recovered from archaeal enrichment cultures. These enzymes, called alkyl-coenzyme M reductase (Acrs), are widespread in the environment but remain poorly understood. Here we produced anoxic cultures degrading mid-chain petroleum *n*-alkanes between pentane (C_5_) and tetradecane (C_14_) at 70 °C using oil-rich Guaymas Basin sediments. In these cultures, archaea of the genus *Candidatus* Alkanophaga activate the alkanes with Acrs and completely oxidize the alkyl groups to CO_2_. *Ca*. Alkanophaga form a deep-branching sister clade to the methanotrophs ANME-1 and are closely related to the short-chain alkane oxidizers *Ca*. Syntrophoarchaeum. Incapable of sulfate reduction, *Ca*. Alkanophaga shuttle electrons released from alkane oxidation to the sulfate-reducing *Ca*. Thermodesulfobacterium syntrophicum. These syntrophic consortia are potential key players in petroleum degradation in heated oil reservoirs.

## Abstract (web summary)

Oil-rich deep-sea sediments are used to culture syntrophic communities of archaea and bacteria that pair petroleum alkane oxidation to sulfide generation.

## Main

In deep seafloor sediments, pressure and heat transform buried organic matter into complex hydrocarbon mixtures, forming natural gas and crude oil^1,2^. *n*-Alkanes (hereafter referred to as ‘alkanes’) constitute a major fraction of these mixtures^3^ and become energy-rich substrates for microorganisms^4^ in habitable anoxic zones. Sulfate-reducing bacteria (SRB) oxidize alkanes ≥ propane (C_3_ alkane)^5,6^ after activation via fumarate addition through alkylsuccinate synthases^7^. Archaea possess a different mechanism for anaerobic alkane degradation based on reversal of the methanogenesis pathway. This mechanism was first revealed in anaerobic methanotrophic archaea (ANME)^8,9^, which activate methane to methyl-coenzyme M (methyl-CoM) via the key enzyme of methanogenesis methyl-coenzyme M reductase (Mcr)^10^. Recently cultured archaea oxidize non-methane alkanes analogously to ANME, as a first step activating the alkanes to alkyl-CoMs via divergent variants of the Mcr, termed alkyl-CoM reductases (Acrs)^11^. *Candidatus* Argoarchaeum ethanivorans^12^, *Ca*. Ethanoperedens thermophilum^13^ and *Ca*. Syntrophoarchaeum spp.^14^ oxidize short-chain gaseous alkanes (C_2_-C_4_), while *Ca*. Methanoliparum spp., enriched from oil-rich environments, oxidize long-chain alkanes (≥C_16_)^15^. Similar to most ANME, the short-chain alkane-oxidizing archaea lack respiratory pathways and shuttle the electrons from alkane oxidation to partner SRB^13,14,16,17^. In contrast, *Ca*. Methanoliparum encodes a canonical Mcr in addition to the Acr, with which it couples alkane oxidation to methanogenesis in a single cell^15^.

Anaerobic archaea capable of petroleum alkane (C_5_-C_15_) oxidation via Acrs were unknown. These alkanes are the major constituents of gasoline and kerosene^18,19^, and of high ecological relevance because of their toxicity^20,21^. Lately, many *acr* genes with unknown function have been recovered from environmental metagenomes, especially from hot springs^22–24^. We hypothesized that yet uncultured thermophilic archaea could activate petroleum alkanes via Acrs. We aimed to enrich such archaea from heated oil-rich sediment from the hydrothermal vent site Guaymas Basin (Gulf of California, Mexico)^25^. We obtained eight enrichment cultures thriving at 70 °C, in which alkanes from C_5_-C_14_ were oxidized in combination with sulfate reduction. Analyses of these cultures via omics approaches and physiological tests revealed that a sister clade of ANME-1, *Ca*. Alkanophaga, was oxidizing the alkanes after activation via Acrs coupled to sulfate reduction by a partner *Thermodesulfobacterium*. Such consortia potentially contribute to souring in deeply buried, heated oil reservoirs.

## Results

### Thermophilic microorganisms thrive on petroleum alkanes

**Fig. 1:**
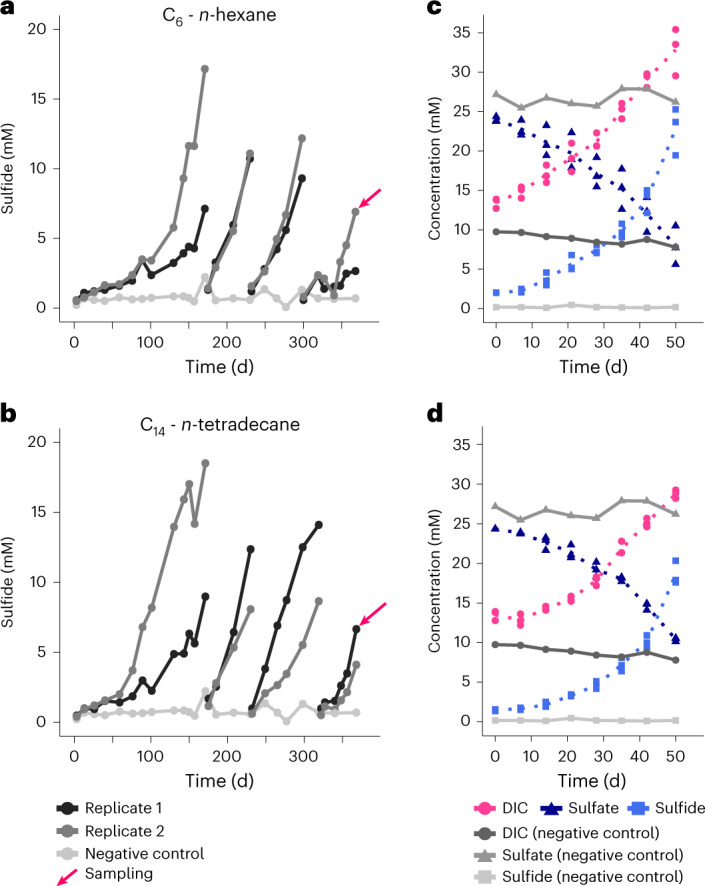
Metabolic activity in anaerobic petroleum alkane-oxidizing cultures at 70 °C. **a**,**b**, Formation of sulfide over time in *n*-hexane (C_6_) (**a**) and *n*-tetradecane (C_14_) (**b**) cultures. Gaps in concentration profiles indicate dilution events. Arrows mark sampling for metagenomic and transcriptomic analyses. **c**,**d**, Concentrations of dissolved inorganic carbon (DIC), sulfate and sulfide in the C_6_ (**c**) and C_14_ (**d**) cultures, and in abiotic controls. For the cultures, three replicate samples were measured, with arithmetic mean shown as a dotted line. Source data

**Extended Data Fig. 1:**
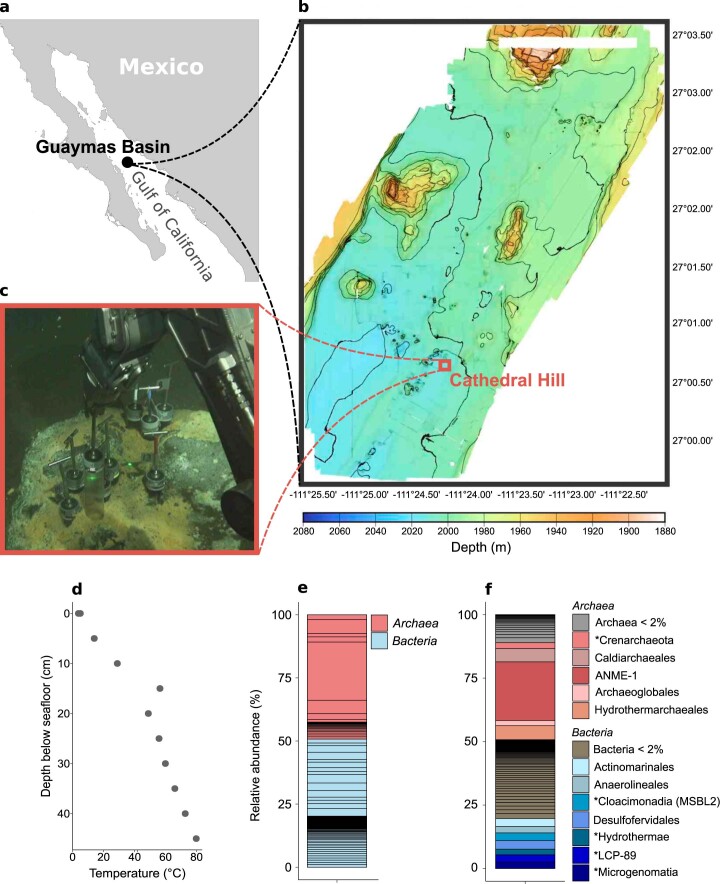
Sampling site in the Guaymas Basin and microbial community in the original sediment. **a**, Location of the Guaymas Basin in the Gulf of California. **b**, Bathymetry of the southern end of the Southern Trough of the Guaymas Basin with the location of the Cathedral Hill hydrothermal vent area. **c**, Sampling of the push core (4991-15) that was used for anoxic cultivations in an area densely covered by orange sulfur-oxidizing *Beggiatoa* mats. **d**, Depth-temperature profile in the sampling site. The temperature was measured using *Alvin*’s heatflow probe. Push cores reached about 30 cm into the sediment, where the temperature approached about 60 °C (sampling site photograph and temperature data courtesy of the Woods Hole Oceanographic Institution, from RV *Atlantis* cruise AT42-05). **e**, **f** Microbial community in the anoxic sediment slurry prepared from core 4991-15 before starting anoxic incubations based on 16S rRNA gene fragments recruited from the metagenome. **e**, On the domain level, archaea and bacteria each make up around 50%. **f**, Taxonomic groups on order level. For groups with unknown order assignment marked with *, the next known higher taxonomic levels are indicated. An ANME-1 group is abundant within the archaeal fraction while the bacterial fraction is very diverse. Source data

**Extended Data Fig. 2:**
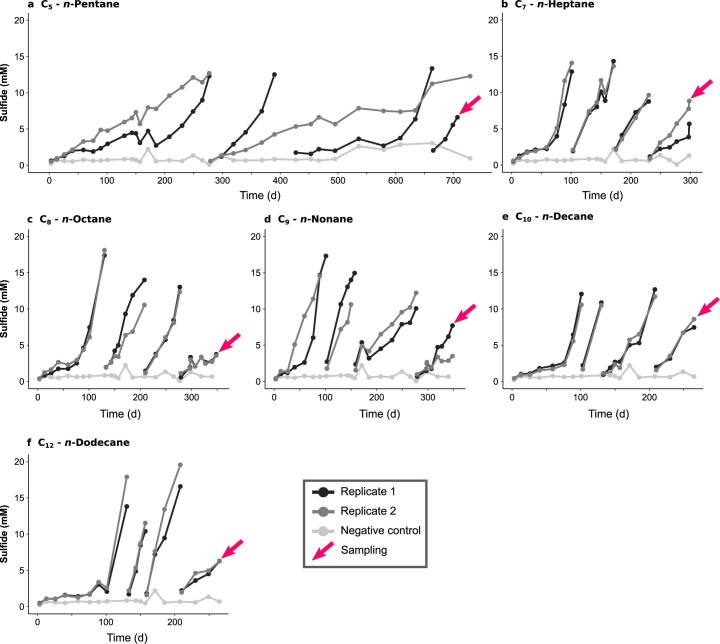
Sulfide production in anoxic C_5_-C_12_*n*-alkane-degrading cultures at 70 °C up to the third dilution. Each culture was set up as a duplicate. Gaps in sulfide level indicate dilution steps. Pink arrows indicate the sampling points for metagenome and -transcriptome sequencing. Samples were collected after the third dilution from cultures degrading (**a**) *n*-pentane, (**b**) *n*-heptane, (**c**) *n*-octane, (**d**) *n*-nonane, (**e)** *n*-decane, and (**f**) *n*-dodecane. The negative control (light gray line) consisted of a sediment slurry without added substrate. Source data

Anoxic slurries produced from heated sediment collected at the hydrothermal vent complex Cathedral Hill in the Southern Trough of the Guaymas Basin (Extended Data Fig. 1a–d) were amended with petroleum alkanes (C_5_-C_14_) as sole carbon and electron source and sulfate as electron acceptor, and incubated at 70 °C. Within 3–7 months, the slurries produced >10 mM sulfide. Sulfide production was accompanied by dissolved inorganic carbon (DIC) production and sustained during dilution steps (Fig. 1 and Extended Data Fig. 2), yielding effectively sediment-free cultures after the third dilution. Cultures, except the considerably slower C_5_ culture, doubled within 13–40 d (Supplementary Table 1).

According to the general formula

1 $$\begin{array}{l}{{\rm{C}}_n}{\rm{H}}_{2n+2}+(0.75n + 0.25){\rm{SO}}_4^{2-} \to n{\rm{HCO}}_3^- + \\ (0.75n+ 0.25){\rm{HS}}^{-}+{\rm{H}}_{2}{\rm{O}}+(0.25n-0.25){\rm{H}}^+,\end{array}$$

the ratio of DIC production to sulfate reduction is ~1.25–1.30 in case of complete alkane oxidation. In two representative cultures (C_6_ and C_14_), this ratio was slightly lower, with 1.21 ± 0.22 in the C_6_ culture and 1.09 ± 0.04 in the C_14_ culture. These values suggest that around 10% (C_6_) and 35% (C_14_) of the carbon released from alkane oxidation is assimilated into biomass (Supplementary Table 2).

### *Ca.* Alkanophagales archaea are abundant in the cultures

**Fig. 2:**
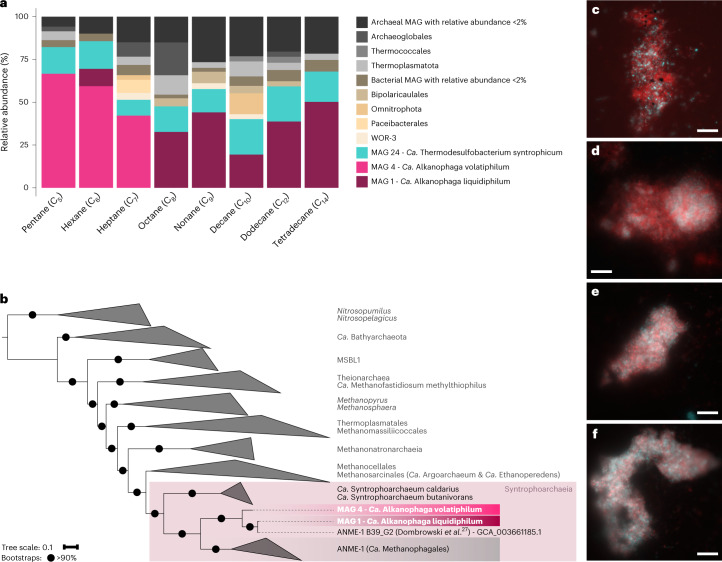
Two archaea of the genus *Ca.* Alkanophaga are abundant in the cultures and closely related to ANME-1. **a**, Relative abundances of MAGs obtained from manual binning. *Ca*. Alkanophaga volatiphilum (MAG 4) is abundant in cultures oxidizing shorter, volatile alkanes between C_5_-C_7_; *Ca*. Alkanophaga liquidiphilum (MAG 1) is abundant in cultures oxidizing liquid alkanes between C_8_ and C_14_. A *Thermodesulfobacterium* with the genomic capacities for dissimilatory sulfate reduction, *Ca*. Thermodesulfobacterium syntrophicum, is present in all cultures. Taxonomies of background MAGs are displayed at order level. Background archaea are shaded grey; background bacteria are shaded brown. **b**, Phylogenomic placement of *Ca*. Alkanophaga MAGs based on the concatenated alignment of 76 archaeal single-copy core genes. *Ca*. Alkanophaga diverge at the root of ANME-1 (*Ca*. Methanophagales). The class Syntrophoarchaeia is highlighted with a shaded rectangle. The outgroup consists of members of the Thermoproteota. Tree scale bar, 10% sequence divergence. **c**–**f**, Double hybridization of C_6_ (**c**,**d**) and C_14_ (**e**,**f**) culture samples with a specific probe targeting the *Ca*. Alkanophagales clade (Aph183, red) and a general bacterial probe (EUBI-III, cyan). *Ca*. Alkanophaga cells are abundant in the aggregates where they co-occur with bacterial cells. Scale bar, 10 µm. Source data

We reconstructed two high-quality archaeal metagenome-assembled genomes (MAGs) from the cultures (Supplementary Table 3): MAG 4, abundant in the C_5_-C_7_ cultures and MAG 1, abundant in the C_8_-C_14_ cultures (Fig. 2a and Supplementary Table 4). Both MAGs were rare (relative abundances ≤0.1%) in the original slurry (Extended Data Fig. 1e,f). The in situ temperatures of the studied sediment (Extended Data Fig. 1d), which captured only the upper sediment layer up to 30 cm depth, probably did not reach the optimal growth temperatures of the two organisms. Both MAGs recruited up to 39% (MAG 4) and 5% (MAG 1) of raw reads in deeper, hotter layers of the Guaymas Basin^26^ (Supplementary Table 5).

MAGs 1 and 4 represent two species within one genus (average nucleotide identity (ANI) 81.5%) and belong to the same genus as the previously published MAG ANME-1 B39_G2 reconstructed from Guaymas Basin sediments (ANIs: MAG 1-ANME-1 B39_G2 98.8% and MAG 4-ANME-1 B39_G2 80.8%)^27^. The name *Ca*. Alkanophagales was recently proposed for the clade represented by ANME-1 B39_G2 on the basis of its genomic content which hinted at a capacity for multicarbon alkane metabolism^11,27^. MAGs 1 and 4 form a clade diverging at the root of ANME-1 and next to *Ca*. Syntrophoarchaeum, together forming the class Syntrophoarchaeia (Fig. 2b).

Visualization of the organisms revealed mixed aggregates of archaea of the *Ca*. Alkanophagales clade and bacteria (Fig. 2c–f). These associations resemble those of short-chain alkane-oxidizing cultures^13,14,16^, suggesting that archaea oxidize the alkanes and partner SRB perform sulfate reduction.

### The enriched archaea activate alkanes with Acrs

**Fig. 3:**
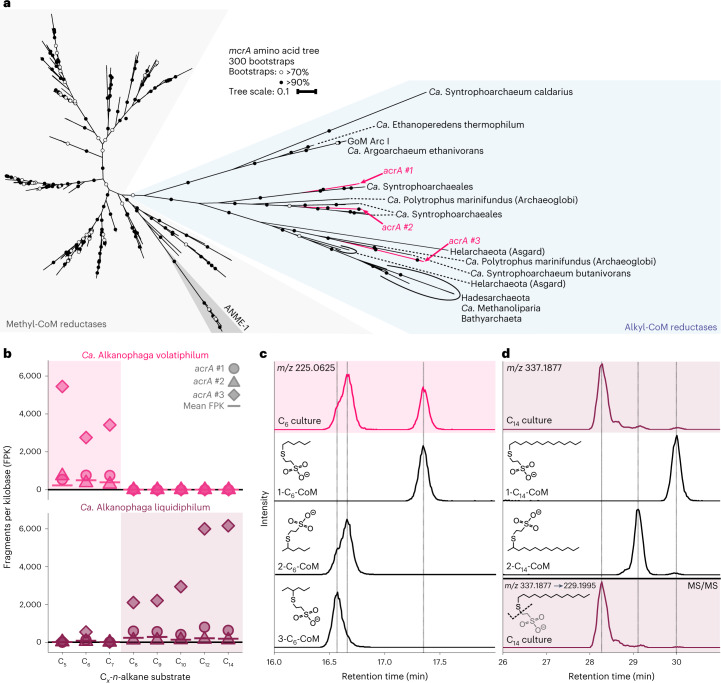
*Ca*. Alkanophaga use alkyl-coenzyme M reductases to activate alkanes to alkyl-CoMs. **a**, Phylogenetic placement of translated *mcrA* sequences of *Ca*. Alkanophaga. Both *Ca*. Alkanophaga species contain three *mcrA* sequences, all of which fall into the divergent branch of *mcrA*s, encoding alkyl-CoM reductases (Acrs), highlighted in blue. The six *acrA* sequences form three clusters of two sequences, each cluster containing one sequence of each *Ca*. Alkanophaga species. Tree scale bar, 10% sequence divergence. **b**, Expression of *acrA* genes during growth on various alkanes for both *Ca*. Alkanophaga species. Cultures in which the respective species was prevalent in the metagenomes are highlighted with shaded boxes. The mean expression of all genes of the respective species is shown as a horizontal bar. The *acrA* of the third cluster was strongly expressed, irrespective of substrate length, by the species abundant in that culture. The expression of the other *acrA* genes was low. **c**,**d**, Extracted ion chromatograms (EICs) based on exact mass and a window of ±10 mDa of deprotonated ions of variants of C_6_-CoM (**c**) and C_14_-CoM (**d**) detected via liquid chromatography–mass spectrometry. In both **c** and **d**, the upper shaded panels show the culture extract, with isomers of alkyl-CoM standards below. In **d**, the shaded bottom panel shows the EIC produced with the exact mass of the C_14_-thiolate, a fragmentation product derived in MS/MS experiments from the precursor C_14_-CoM. Dashed vertical lines were added at retention times of peak maxima of standards (**c**) or standards and fragmentation products (**d**) for easier identification of peaks in the culture extracts. While C_6_ is activated on the first and second carbon atom to a similar degree, C_14_ is activated predominantly to ≥3-C_14_-CoM. Source data

**Extended Data Fig. 3:**
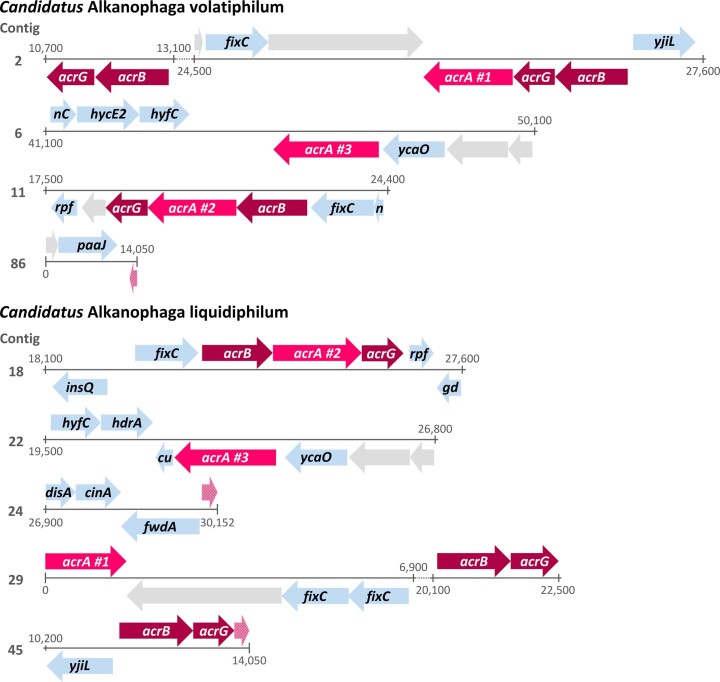
Organization of *acr* genes in *Candidatus* Alkanophaga MAGs. Partial *acrA* genes are shown in light pink, unannotated genes in light gray. Some gene names were shortened to fit the arrows. Genes code for: *acrA*: alkyl-coenzyme M reductase, alpha subunit; *acrB*: alkyl-coenzyme M reductase, beta subunit; *acrG*: alkyl-coenzyme M reductase, gamma subunit; *fixC*: flavoprotein dehydrogenase; *yjiL*: activator of 2-hydroxyglutaryl-CoA dehydratase; *nC*: *nuoC*-NADH:ubiquinone oxidoreductase; *hycE2*: [NiFe]-hydrogenase III large subunit; *hyfC*: formate hydrogenlyase; *ycaO*: ribosomal protein S12 methylthiotransferase accessory factor; *rpf*: *rpf1*-rRNA maturation protein; *n*: *nuoI*-formate hydrogenlyase subunit 6; *paaJ*: acetyl-CoA acetyltransferase; *insQ*: transposase; *gd*: *gdb1*-glycogen debranching enzyme; *hdrA*: heterodisulfide reductase, subunit A; *cu*: *cutA1*-divalent cation tolerance protein; *disA*: c-di-AMP synthetase; *cinA*: ADP-ribose pyrophosphatase domain of DNA damage- and competence-inducible protein CinA; *fwdA*: formylmethanofuran dehydrogenase subunit A. Source data

Both *Ca*. Alkanophagales MAGs encode three Acrs (*acrABG*) (Extended Data Fig. 3). Currently, only the sister group *Ca*. Syntrophoarchaeum encodes a higher number of Acrs with four copies^14^. The six *acrA* sequences, which code for the catalytic subunit^28^, form three clusters of two highly similar sequences, one of each species (≥89% identity) in the *acrA* clade (Fig. 3a and Supplementary Table 6)^12–15^. All clusters are highly similar to *acrAs* of *Ca*. Syntrophoarchaeum (Supplementary Table 6).

Both species highly expressed the *acrA* of the third cluster, placing it among the top 19 (C_8_) to top 4 (C_5_) expressed genes (Fig. 3b and Supplementary Table 7). This cluster is phylogenetically closely related to *acrA*s that presumably activate long-chain alkanes, for instance in *Ca*. Methanoliparum^15^. In both MAGs, this *acrA* is spatially separated from the *acrB* and *acrG* subunits (Extended Data Fig. 3), which has been previously reported for *Ca*. Syntrophoarchaeum^14^.

**Extended Data Fig. 4:**
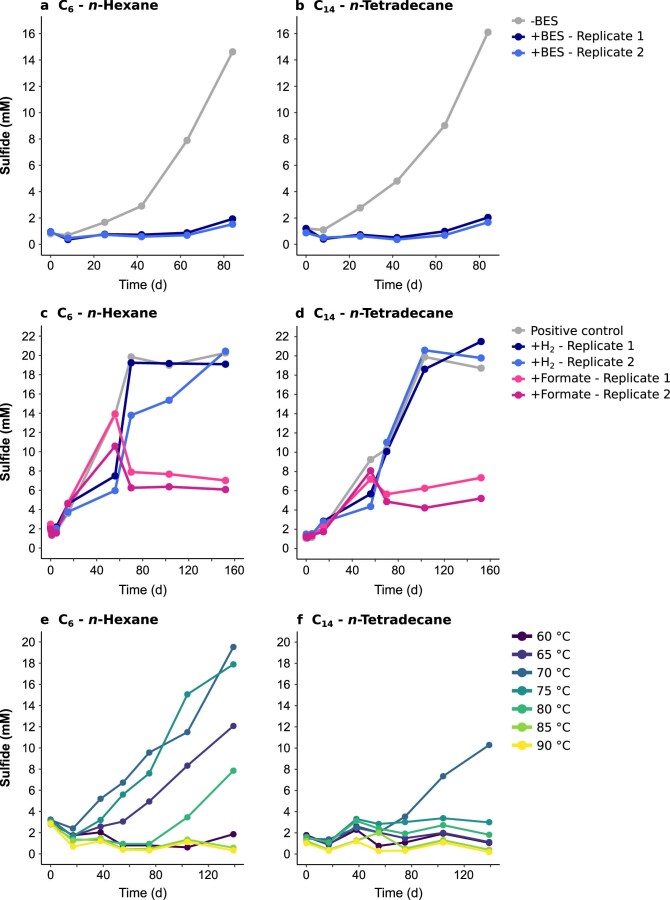
Sulfide production in *n*-hexane (C_6_)- and *n*-tetradecane (C_14_)-degrading cultures under different conditions. **a, b**, Treatment with 2-bromoethanosulfonate (BES). BES (5 mM final concentration) was added to duplicates of C_6_ (**a**) and C_14_ (**b**) degrading cultures ( + BES). A control culture (-BES) did not receive BES. The inhibition of alkane oxidation by BES corroborates an Acr-based substrate activation. **c,d**, Addition of hydrogen or formate to C_6_ (**c**) and C_14_ (**d**)-degrading cultures. All cultures were supplied with the original substrate. The addition of 10% H_2_ into the headspace or 10 mM sodium formate into the medium did not accelerate sulfide production compared to positive controls. **e, f**, Incubation at temperatures between 60 °C and 90 °C. The C_6_-degrading culture (**e**) grows optimally at 70 °C and 75 °C, while it still shows some activity at slightly lower (65 °C) and slightly higher (80 °C) temperatures. The activity of the C_14_-degrading culture (**f**) seems to be limited to 70 °C. Source data

**Extended Data Fig. 5:**
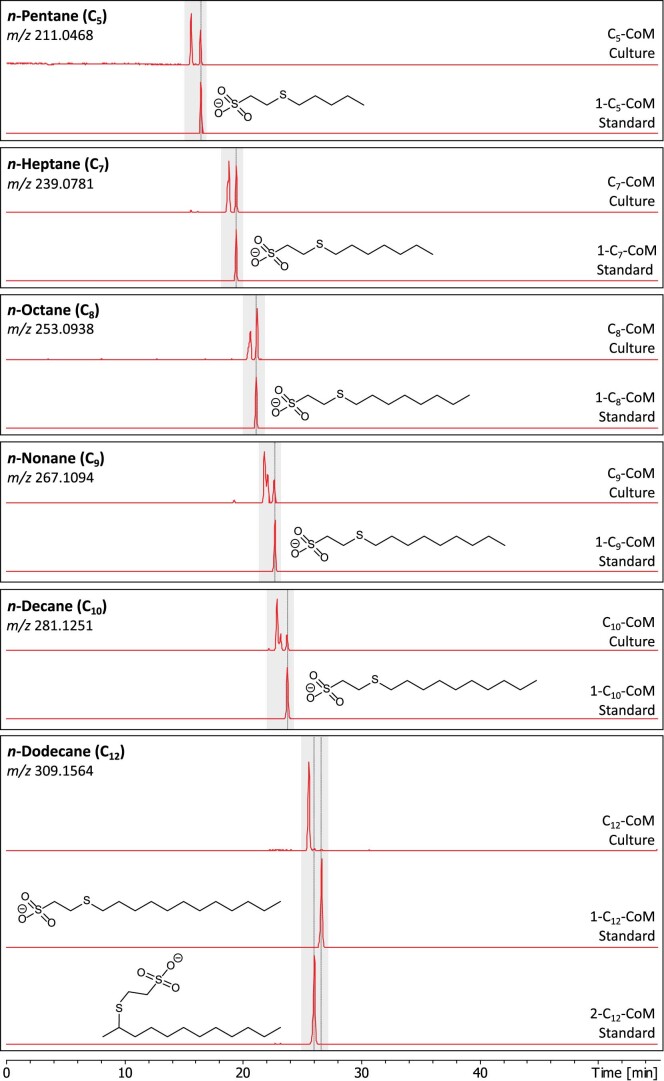
Detection of alkyl-CoMs in C_x_-*n*-alkane-degrading cultures. Samples were separated by liquid chromatography and extracted ion chromatograms (EICs) based on the exact mass of deprotonated ions of the C_x_-alkyl-CoMs with a window of ±10 mDa were created. Panels show the EICs of culture extracts together with synthetic standards. Dashed vertical lines were added at the retention times of peak maxima of the standards for easier identification of peaks in the culture extracts. Peaks with mass-to-charge ratios (*m/z*) of the respective alkyl-CoM were detected in all cultures. All culture extracts show several peaks, indicating an activation at different carbon atoms. While shorter alkanes are activated to a similar degree at subterminal and terminal positions, longer alkanes are predominantly activated at non-terminal carbon atoms.

As in other Acr-dependent alkane-degrading cultures^14^^,29^, a selective inhibitor of the Mcr/Acr, the CoM analogue 2-bromoethanosulfonate (BES)^30^, suppressed sulfide production (Extended Data Fig. 4a,b), consistent with an Acr-based activation mechanism. Further, metabolite extracts of all cultures contained peaks pertaining to the masses of the corresponding alkyl-CoMs as indicative activation product (Fig. 3c,d and Extended Data Fig. 5). While alkanes from C_5_-C_7_ were activated at the first and second carbon atom in similar ratios (Fig. 3c and Extended Data Fig. 5), we observed a shift to more subterminally activated alkanes with increasing alkane length (≥C_9_) (Fig. 3d and Extended Data Fig. 5). The longest alkanes C_12_ and C_14_ seemed to be activated predominantly to ≥3-alkyl-CoM (Fig. 3d and Extended Data Fig. 5). An activation at multiple positions was previously observed in *Ca*. Syntrophoarchaeum^14^. The comparatively high activation rate at the terminal position for shorter alkanes is unexpected, because particularly in short alkanes, C-H bonds are stronger at terminal positions compared with subterminal positions^31^. Further degradation of non-terminally activated alkanes probably requires a rearrangement to 1-alkyl-CoM as described for bacterial alkane degradation^32^.

**Extended Data Fig. 6:**
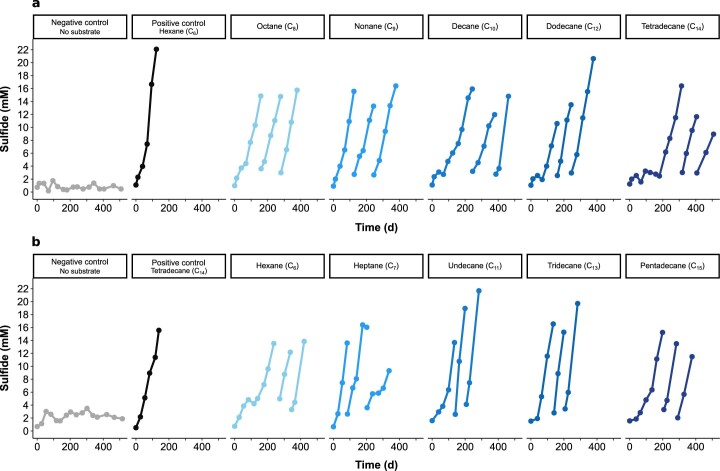
Substrate spectra of originally (a) *n*-hexane- and (b) *n*-tetradecane-oxidizing enrichment cultures. Cultures were diluted into fresh sulfate-reducer medium and supplemented with other *n*-alkanes between C_3_ and C_20_. Only active cultures are shown. No activity was observed for cultures supplied with C_3_, C_4_, C_16_, C_18_, or C_20_. Source data

We conclude that the archaea represented by MAGs 1 and 4 oxidize the petroleum alkanes. We propose the genus name *Ca*. Alkanophaga, consistent with the previously suggested name *Ca.* Alkanophagales^11^, and analogous to the closely related methanotrophs *Ca*. Methanophagales (ANME-1)^33^. The *Ca*. Alkanophaga MAGs share amino acid identities (AAIs) of 55–59% with ANME-1 MAGs (Supplementary Table 8), placing *Ca*. Alkanophaga within the ANME-1 family^34^. On the basis of apparent substrate preference in our enrichment cultures, we propose the names *Ca*. Alkanophaga volatiphilum for the archaeon represented by MAG 4 and *Ca*. Alkanophaga liquidiphilum for the archaeon represented by MAG 1. Substrate tests corroborate that *Ca*. A. volatiphilum prefers shorter volatile alkanes <C_10_, while *Ca*. A. liquidiphilum readily degrades all alkanes between C_6_ and C_15_ (Extended Data Fig. 6).

### *Ca*. Alkanophaga completely oxidize the alkanes to CO_2_

**Fig. 4:**
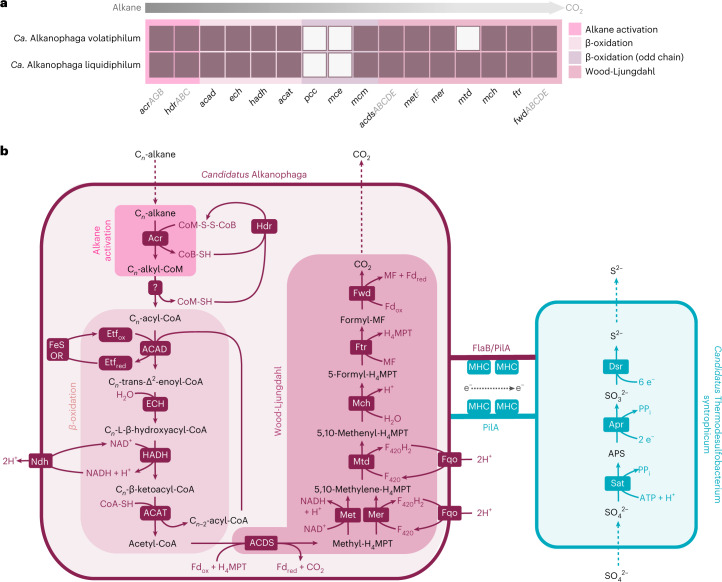
Mechanism of syntrophic petroleum alkane oxidation. **a**, Genomic capacities for alkane oxidation in *Ca.* Alkanophaga MAGs. Colour-filled rectangles indicate presence of a gene; white rectangles indicate absence. For multiple-subunit proteins, at least one gene coding for each subunit was found in case of a filled rectangle. **b**, Metabolic model for syntrophic alkane oxidation. *Ca*. Alkanophaga activates alkanes via the alkyl-coenzyme M reductase (Acr). A yet unknown pathway transforms alkyl-CoM to acyl-CoA. The enzymes of the β-oxidation pathway, including (1) acyl-CoA dehydrogenase (ACAD), (2) enoyl-CoA hydratase (ECH), (3) hydroxyacyl-CoA dehydrogenase (HADH) and (4) acyl-CoA acetyltransferase (ACAT), cleave acyl-CoA into multiple acetyl-CoA units. The acetyl-CoA decarbonylase/synthase (ACDS) complex breaks the acetyl units into CO_2_ and a tetrahydromethanopterin (H_4_MPT)-bound methyl unit. The methyl branch of the Wood-Ljungdahl pathway, including (1) 5,10-methylene tetrahydrofolate reductase (MetF) and/or 5,10-methylene H_4_MPT reductase (Mer), (2) methylene-H_4_MPT dehydrogenase (Mtd), (3) methenyl-H_4_MPT cyclohydrolase (Mch), (4) formylmethanofuran-H_4_MPT formyltransferase (Ftr) and (5) tungsten-containing formylmethanofuran dehydrogenase (Fwd), oxidizes methyl-H_4_MPT to CO_2_. Most probably, an electron transfer flavoprotein (Etf) serves as electron acceptor in the first step of the β-oxidation pathway. Cofactor recycling is taken over by cytoplasmic heterodisulfide reductase (Hdr), [FeS]-oxidoreductase (FeS-OR), NADH dehydrogenase (Ndh) and F_420_H_2_:quinone oxidoreductase (Fqo). Electrons from alkane oxidation are transferred to *Ca*. Thermodesulfobacterium syntrophicum, most probably via DIET. DIET seems to rely on conductive filaments formed by type IV pilin (PilA) and/or flagellin B (FlaB) that are expressed by both partners, and multihaem *c*-type cytochromes (MHCs) expressed solely by the bacterium. Sulfate reduction in *Ca*. T. syntrophicum follows the canonical dissimilatory sulfate pathway using the enzymes ATP-sulfurylase (Sat), APS-reductase (Apr) and dissimilatory sulfite reductase (Dsr). *pcc*, gene encoding propionyl-CoA decarboxylase; *mce*, gene encoding methylmalonyl-CoA epimerase.

The oxidation of alkyl-CoMs generated by the Acr requires conversion to acyl-CoA (Fig. 4a,b). The underlying reactions for this transformation are unknown, but for other alkane-degrading archaea, some candidate enzymes have been proposed. The C_2_-oxidizing *Ca*. Ethanoperedens thermophilum may catalyse this step with tungstate-containing aldehyde:ferredoxin reductases (Aors). This archaeon encodes three *aor* copies located closely to genes of the Wood-Ljungdahl (WL) pathway and expresses them during ethane oxidation^13^. While both *Ca*. Alkanophaga encode complete *aor* gene sets, those genes were only moderately expressed (Supplementary Table 7), casting doubt on a crucial role of the Aor in this reaction in our cultures. A transfer of alkyl moieties to CoA via methyltransferases, as was hypothesized for *Ca*. Syntrophoarchaeum^14^, is equally unlikely because of the large alkanes consumed by *Ca*. Alkanophaga. In conclusion, the conversion of alkyl-CoM to acyl-CoA requires further investigation.

**Fig. 5:**
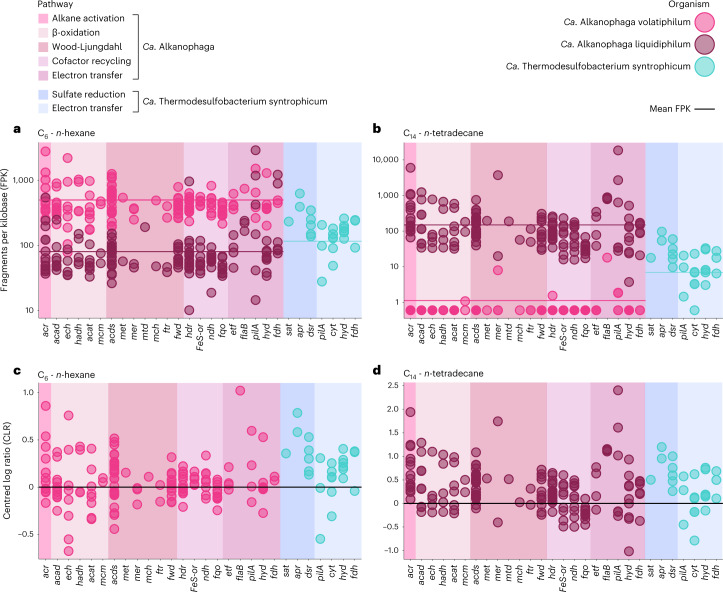
Gene expression profiles for syntrophic petroleum alkane oxidation. **a**,**b**, Fragment counts normalized to gene length (FPK) shown on a logarithmic *y* axis. The average gene expression of each organism is indicated as arithmetic mean (sum of all FPK values divided by number of genes) depicted as a horizontal line. **c**,**d**, Fragment counts normalized as CLR. For simplicity, only the values of the more active *Ca*. Alkanophaga species are shown. For abbreviations, see Fig. 4; *hyd*, gene encoding [NiFe]-hydrogenase; *fdh*, gene encoding formate dehydrogenase; *cyt*, gene encoding multihaem cytochrome. Source data

**Extended Data Fig. 7:**
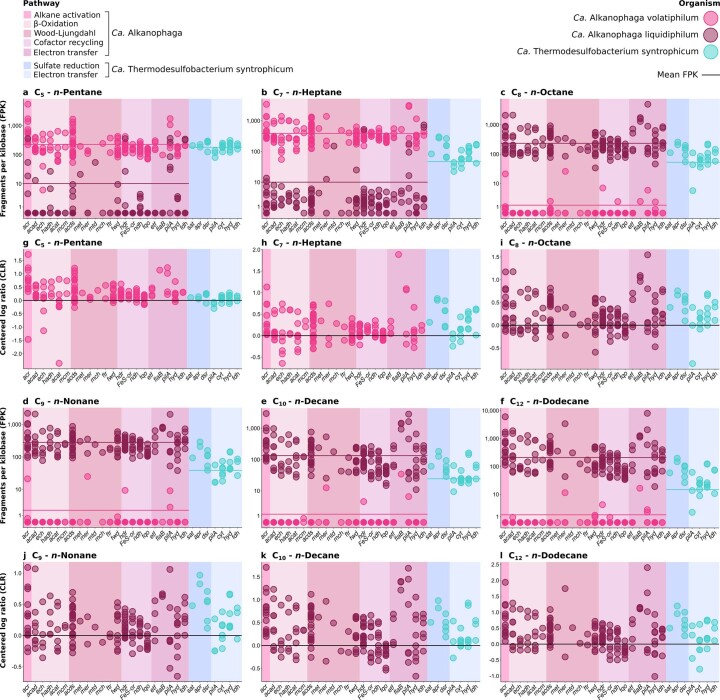
Expression of alkane oxidation, sulfate reduction, and related genes in C_5_-C_12_*n*-alkane-oxidizing cultures. Transcriptome reads were mapped to the MAGs of the two *Candidatus* Alkanophaga species and to *Ca*. Thermodesulfobacterium syntrophicum. **a-f**, Fragment counts normalized to gene length (FPK) using a logarithmic y axis. The average gene expression of each organism is indicated as arithmetic mean (sum of all FPK values divided by number of genes) depicted as a horizontal line. **g-l**, Fragment counts normalized as CLR. For simplicity, only values of the more active *Ca*. Alkanophaga species are shown. The x-axis shows the genes encoding: *acr*: alkyl-CoM reductase, *acad*: acyl-CoA dehydrogenase, *ech*: enoyl-CoA hydratase, *hadh*: hydroxyacyl-CoA dehydrogenase, *acat*: acetyl-CoA acetyltransferase, *mcm*: methylmalonyl-CoA mutase, *acds*: acetyl-CoA decarbonylase/synthase, *met*: 5,10-methylene tetrahydrofolate reductase, *mer*: 5,10-methylene tetrahydromethanopterin (H_4_MPT) reductase, *mtd*: methylene-H_4_MPT dehydrogenase, *mch*: methenyl-H_4_MPT cyclohydrolase, *ftr*: formylmethanofuran-H_4_MPT formyltransferase, *fwd*: tungsten-containing formylmethanofuran dehydrogenase, *hdr*: heterodisulfide reductase, *FeS-or*: [FeS]-oxidoreductase, *ndh*: NADH dehydrogenase, *fqo*: F_420_H_2_:quinone oxidoreductase, *etf*: electron transfer flavoprotein, *flaB*: flagellin B, *pilA*: type IV pilin, *hyd*: [NiFe]-hydrogenase, *fdh*: formate dehydrogenase, *sat*: ATP-sulfurylase, *apr*: APS-reductase, *dsr*: dissimilatory sulfite reductase, *cyt*: multi-heme *c*-type cytochrome. Source data

Similar to *Ca*. Syntrophoarchaeum^14^, *Ca*. Alkanophaga probably processes acyl-CoA to acetyl-CoA units via the β-oxidation pathway^35^ (Fig. 4b). *Ca*. Alkanophaga encode all genes for even-chain β-oxidation and expressed them during alkane oxidation (Figs. 4a and 5, Extended Data Fig. 7 and Supplementary Table 7). For odd-chain alkanes, three additional genes are required to degrade the potentially toxic C_3_-compound propionyl-CoA^36,37^, two of which are missing from *Ca*. Alkanophaga. We could not identify complete alternative pathways for the degradation of propionyl-CoA, for example the methylcitrate cycle^37^. Thus, the fate of the propionyl-CoA remains, for the moment, unclear.

Acetyl-CoA units from β-oxidation are shuttled into biomass production or completely oxidized. For the latter, the acetyl-CoA decarbonylase/synthase (ACDS) complex splits a methyl group from acetyl-CoA which is transferred to tetrahydromethanopterin (H_4_MPT) (Fig. 4b). The enzymes of the H_4_MPT methyl branch of the WL pathway then oxidize methyl-H_4_MPT to CO_2_^13,14^. Both *Ca*. Alkanophaga species encode and expressed multiple ACDS and all enzymes of the WL pathway, except methylene-H_4_MPT-deyhdrogenase (*mtd*) missing in *Ca*. A. volatiphilum (Figs. 4a and 5, Extended Data Fig. 7 and Supplementary Table 7).

**Extended Data Fig. 8:**
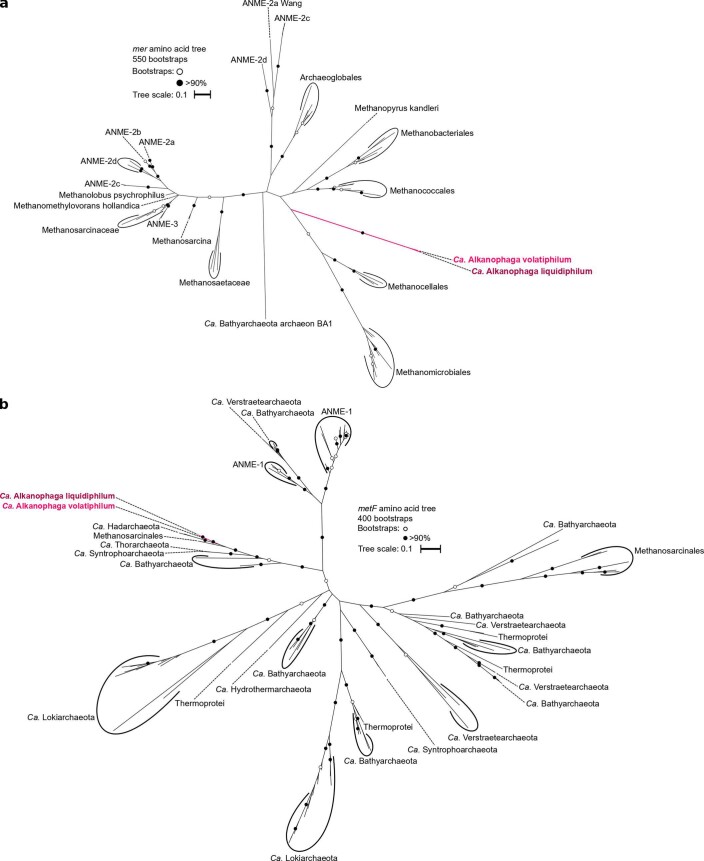
Phylogenetic placement of (a) 5,10-methylene-H_4_MPT reductase (*mer*) and (b) methylenetetrahydrofolate reductase (*metF*) sequences recovered from *Ca*. Alkanophaga MAGs. Both *mer* and *metF* sequences of the two *Ca*. Alkanophaga species are highly similar to each other. The *mer* sequences, which distinguish *Ca*. Alkanophaga in the class Syntrophoarchaeia, might originate from the ancestor of Methanocellales, while *metF* sequences cluster near those of close relatives *Ca*. Syntrophoarchaeales. Source data

Unlike the closely related *Ca*. Syntrophoarchaeum and ANME-1, both *Ca*. Alkanophaga encode several 5,10-methylene-H_4_MPT reductase (*mer*) genes. This enzyme catalyses the oxidation of methyl-H_4_MPT (CH_3_-H_4_MPT) to methylene-H_4_MPT (CH_2_ = H_4_MPT) in the first step of the oxidative WL pathway^38^. Two of these genes, OD814_001315 in *Ca*. A. volatiphilum and OD815_000385 in *Ca*. A. liquidiphilum, most probably code for a canonical *mer* because they are highly similar (>99%) to *mer* copies of Methanomicrobia. A phylogenetic analysis placed these two *mer* sequences next to each other and close to those of the hydrogenotrophic methanogens Methanocellales^39^ (Extended Data Fig. 8a). We therefore hypothesize that *Ca*. Alkanophaga inherited *mer* vertically from the methanogenic ancestor of Methanocellales. *Ca*. Syntrophoarchaeum and ANME-1 seem to have replaced *mer* with methylene-tetrahydrofolate (H_4_F) reductase (*metF*) of the H_4_F methyl branch of the WL pathway^14,40^. Both *Ca*. Alkanophaga MAGs also encode *metF* copies, which are highly similar (70–80%) to those of *Ca*. Syntrophoarchaeum and cluster next to *metF* sequences of Hadarchaeota from Jinze hot spring (China) and Yellowstone National Park (USA) (Extended Data Fig. 8b). While both *mer* and *metF* were transcribed, *mer* was especially expressed by *Ca*. A. liquidiphilum in cultures oxidizing longer alkanes ≥C_10_ (Fig. 5b,d, Extended Data Fig. 7e,f,k,l and Supplementary Table 7).

### *Ca*. Alkanophaga partner with a *Thermodesulfobacterium*

**Extended Data Fig. 9:**
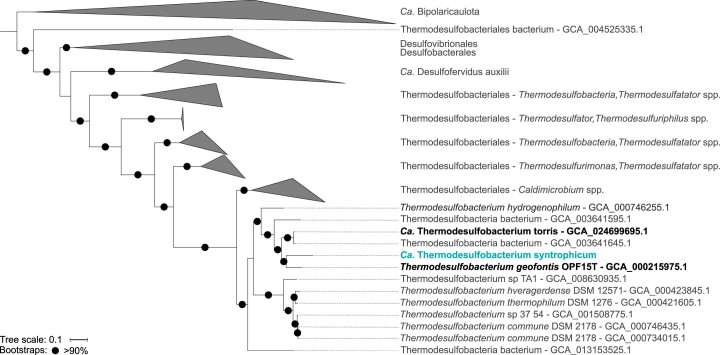
Phylogenomic placement of *Candidatus* Thermodesulfobacterium syntrophicum based on the concatenated alignment of 71 bacterial single copy core genes. *Ca*. T. syntrophicum is closely related to the already cultured *Thermodesulfobacterium geofontis* (OPF15T) and to *Ca*. Thermodesulfobacterium torris, which functions as partner bacterium in the thermophilic anaerobic oxidation of methane. The outgroup consists of members of the candidate phylum Bipolauricaulota. The tree scale bar indicates 10% sequence divergence. Source data

*Ca*. Alkanophaga lack the dissimilatory sulfate reduction (DSR) pathway and therefore require a partner organism. We identified a *Thermodesulfobacterium* represented by MAG 24, which was enriched in all cultures (Fig. 2a and Supplementary Table 4) and rare in the original slurry, as the most likely syntrophic sulfate reducer. MAG 24 encodes and expressed the three DSR proteins ATP-sulfurylase (Sat), APS-reductase (Apr) and dissimilatory sulfite reductase (Dsr)^41^ (Fig. 5, Extended Data Fig. 7 and Supplementary Table 7). We propose the name *Ca.* Thermodesulfobacterium syntrophicum for this bacterium, which is closely related to the hyperthermophilic sulfate-reducing *Thermodesulfobacterium geofontis* isolated from the Obsidian Pool in Yellowstone National Park (USA)^42^ (Extended Data Fig. 9).

#### Etymology

Alkanophaga: *alkano* (new Latin): alkane and *phaga* (Greek): eating; volatiphilum: *volatilis* (Latin): volatile and *philum* (Greek): preferring; liquidiphilum: *liquidus* (Latin): liquid and *philum* (Greek): preferring; syntrophicum: *syn* (Greek): together with; *trephein* (Greek): nourish and *icum* (Latin): pertaining to.

#### Locality

Hydrothermally heated oil-rich deep-sea sediment in the Guaymas Basin, Gulf of California, Mexico.

#### Description

*Ca.* Alkanophaga volatiphilum and *Ca.* Alkanophaga liquidiphilum: thermophilic, anaerobic, petroleum (C_5_-C_14_) *n*-alkane-oxidizing archaea, forming syntrophic consortia with the sulfate-reducing *Ca.* Thermodesulfobacterium syntrophicum.

Syntrophic microorganisms trade electrons via molecular intermediates, such as hydrogen or formate^43^, or direct interspecies electron transfer (DIET)^44^. Both *Ca*. Alkanophaga and *Ca*. T. syntrophicum encode membrane-bound [NiFe]-hydrogenases, including several hydrogenase maturation factors, enabling electron transfer via molecular hydrogen. Some hydrogenase genes were substantially expressed (Fig. 5, Extended Data Fig. 7 and Supplementary Table 7). Formate dehydrogenases, necessary for electron transfer via formate, were also present in both partners and moderately expressed (Fig. 5, Extended Data Fig. 7 and Supplementary Table 7). However, the addition of hydrogen or formate did not accelerate sulfide production (Extended Data Fig. 4c,d). Moreover, cultures in which sulfate reduction was inhibited by the addition of sodium molybdate produced only miniscule fractions (max. 2.4% for C_6_ and 0.9% for C_14_) of the hydrogen concentrations that would be necessary were hydrogen the sole electron carrier (Supplementary Table 9). Thus, neither molecular hydrogen nor formate are probably primary electron carriers.

Alternatively, alkane oxidation and sulfate reduction are coupled through DIET, as suggested for other alkane-oxidizing consortia^13,14,45^. DIET probably involves cell appendages, such as bacterial type IV pilin (PilA) or the archaeal flagellin B (FlaB), and multihaem *c*-type cytochromes (MHCs), forming conductive nanowires enabling electron transport^46,47^. Both components are present and strongly expressed in previously established alkane-oxidizing consortia^13,14,45^. Surprisingly, neither our nor the previously published *Ca*. Alkanophaga MAGs encode any MHCs, while the closest relatives of *Ca*. Alkanophaga, ANME-1 and *Ca*. Syntrophoarchaeum, encode multiple MHCs^14,33^. *Ca*. T. syntrophicum encodes six MHCs, only one of which was slightly enriched in all cultures (Supplementary Table 7). This implies a minor role of MHCs in the interaction of both organisms.

**Extended Data Fig. 10:**
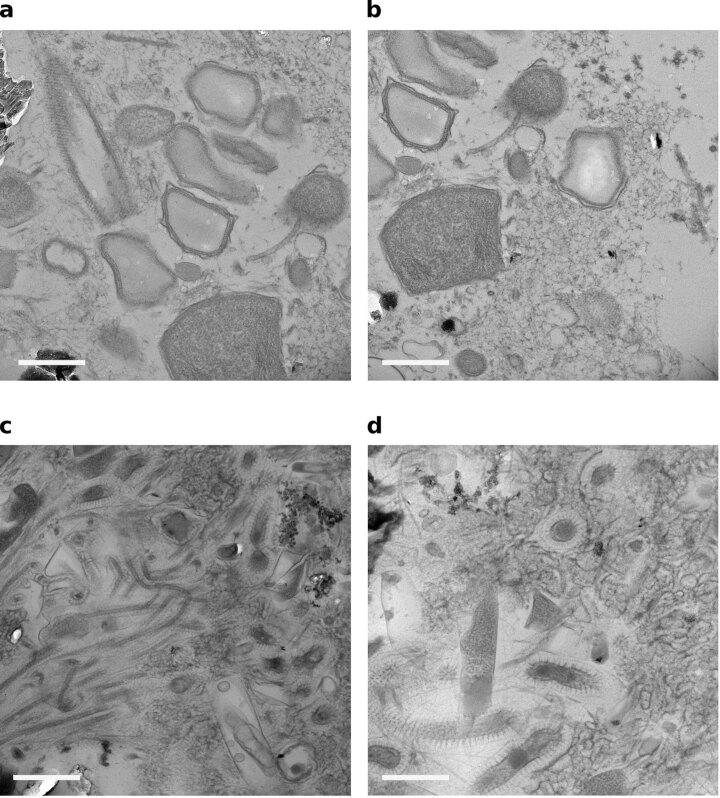
Transmission electron micrographs of EPON 812-embedded thin-sections of (a,b) C_6_- and (c,d) C_14_-*n*-alkane-degrading culture samples. The scale bar indicates 0.5 µm. The experiment was run once with one biological replicate per sample. Images are representative for > 5 recorded images per sample.

Both *Ca*. Alkanophaga encode several copies of *pilA* and *flaB* for the formation of cell appendages for DIET. These genes were among the most highly expressed genes of *Ca*. Alkanophaga in all cultures. *Ca*. T. syntrophicum encodes several *pilA* genes as well, some of which were strongly enriched in the C_10_-C_14_ cultures (Supplementary Table 7). Transmission electron microscopy revealed diffuse filamentous structures in the intercellular space that might pertain to such nanowires (Extended Data Fig. 10), but further analyses are necessary to confirm the identity of these structures.

We predict that electron transfer in our cultures is based predominantly on DIET. The lack of MHCs in *Ca*. Alkanophaga might be compensated by MHC production in the partner bacterium similar to observations in syntrophic methane-oxidizing cultures, where only the bacterial partner expressed *pilA* genes^45^. Alternatively, DIET might be completely independent of MHCs, which has been observed before^48,49^. It remains possible that a small fraction of electrons are transferred via soluble intermediates such as hydrogen. Such a combination of DIET with diffusion-based electron transport was recently shown to be energetically favourable for syntrophic consortia^50^.

## Discussion

Petroleum-rich anoxic environments such as oil reservoirs, oily sludges and polluted sediments harbour oil-degrading microorganisms. Isolates from these environments that couple petroleum alkane oxidation to sulfate reduction are mostly bacteria active at temperatures ≤60 °C (ref. ^51^). With *Ca*. Alkanophaga, we enriched a thermophilic clade thriving on petroleum alkanes from C_5_ to C_14_ at temperatures between 65–75 °C (Extended Data Fig. 4e,f), which approach the suggested upper limit of microbial hydrocarbon degradation in petroleum reservoirs of around 80 °C (ref. ^52^). This temperature optimum is reflected by the high relative abundance of *Ca*. Alkanophaga in deep, heated sediment layers of the Guaymas Basin, inferring a crucial role of these archaea in thermophilic hydrocarbon transformation.

*Ca*. Alkanophaga encode three Acrs for anaerobic alkane activation, one less than the closely related short-chain alkane oxidizer *Ca*. Syntrophoarchaeum^14^. Independent of alkane length, *Ca*. Alkanophaga strongly expressed only one of the Acrs, which is highly similar to the highest expressed Acr in *Ca*. Syntrophoarchaeum during C_4_ oxidation^14^. Future studies may reveal functions or substrates of the other two lower expressed Acrs. *Ca*. Alkanophaga stand out among Acr-using archaea with their wide substrate range between C_5_ and C_15_. Therewith, all alkanes between C_1_ and C_20_ are confirmed substrates of alkane-oxidizing archaea^12–15^. Our study implies that substrate flexibility of the Acr increases with increasing alkane length, which is presumably enabled by a wider catalytic cleft in the Acrs activating C_3_+ alkanes^31^ compared with the highly selective hydrophobic tunnel detected in the C_2_-activating Acr^29^. Crystallization efforts may resolve molecular and structural modifications of these Acrs that make use of such a wide substrate spectrum.

The three clades of the class Syntrophoarchaeia (*Ca*. Alkanophaga, *Ca*. Syntrophoarchaeum and ANME-1), share many metabolic features such as obligate syntrophic growth with partner SRB and presence of the β-oxidation and WL pathways. At the same time, they exhibit remarkable metabolic and genomic differences. For instance, ANME-1 encode the canonical Mcr for methane metabolism, which is missing in *Ca*. Syntrophoarchaeum and *Ca*. Alkanophaga, preventing them from oxidizing and producing methane. Instead, the latter two possess multiple multicarbon alkane-activating Acrs, which are in turn absent in ANME-1. Our study supports the previously established hypothesis that multicarbon alkane metabolism probably preceded methanotrophy in the Syntrophoarchaeia^11,53^ because of the basal position of both multicarbon alkane oxidizers (Fig. 2b) and their similar metabolisms. The presence of the β-oxidation pathway in ANME-1 (ref. ^11^) supports this notion because this pathway is required for the oxidation of C_3_+ alkanes but serves no purpose in the oxidation of methane. We propose that the common ancestor of the Syntrophoarchaeia was a multicarbon alkane-oxidizing archaeon with multiple Acrs. *Ca*. Syntrophoarchaeum and *Ca*. Alkanophaga emerged from this ancestor, preserving a similar metabolism. Today, *Ca*. Syntrophoarchaeum thrives at much lower temperatures (50 °C) and seems incapable of oxidizing liquid alkanes^14^. Thus, adaptation to different temperatures and substrates might have enabled *Ca*. Syntrophoarchaeum and *Ca*. Alkanophaga to occupy different ecological niches. *Ca*. Alkanophaga and ANME-1 also shared a common ancestor from which ANME-1 probably diverged after losing their Acrs^54^ and acquiring an Mcr, potentially from a methanogen via lateral gene transfer^33,55^.

*Ca*. Alkanophaga differ from the two other groups of the Syntrophoarchaeia in two main aspects. First, *Ca*. Alkanophaga encode and expressed *mer*, an essential enzyme of the canonical methanogenesis pathway^56^. ANME-1, except for a putative methanogenic ANME-1 member^57^, and *Ca*. Syntrophoarchaeum lack *mer* and instead code for the phylogenetically widely distributed *metF*^14,33,58^, which is also present and expressed in *Ca*. Alkanophaga. We hypothesize that *mer* in *Ca*. Alkanophaga is a remnant from a methanogenic ancestor. Second, *Ca*. Alkanophaga lack MHCs, which are often considered essential for DIET between syntrophic partners^46^. All other syntrophic alkane-oxidizing archaea code for several MHCs^53^. However, an absence of MHCs in DIET-performing methanogens has been recognized before^48^. It is thus conceivable that MHCs aid in but are not essential for DIET and that MHCs were potentially lost by *Ca*. Alkanophaga without a substantial impact on the efficiency of electron transfer. The loss of all MHCs opens up questions as to the mechanisms that occurred. In a recent study, giant extrachromosomal elements named Borgs, many of which carried clusters of MHCs, were reconstructed from methane-oxidizing *Methanoperedens* (ANME-2d) archaea^59^. One could imagine that MHCs in the Syntrophoarchaeia ancestor were encoded on such a Borg, which was then lost by *Ca*. Alkanophaga. This could explain why all MHCs are absent in *Ca*. Alkanophaga. However, the presence of Borgs in other members of the Syntrophoarchaeia still needs to be examined.

*Ca*. Alkanophaga partner with the sulfate-reducing *Ca*. Thermodesulfobacterium syntrophicum. Previously enriched alkane-oxidizing archaea partner with a different bacterium, *Ca*. Desulfofervidus auxilii, which has an optimal growth temperature of 60 °C (refs. ^13,14,16,17^). We suspect that the higher incubation temperature of our study selected for a more thermophilic partner organism. Recently, another *Thermodesulfobacterium* species, *Ca*. Thermodesulfobacterium torris (ANI 84.0%, Extended Data Fig. 9), has been reported as syntrophic sulfate reducer partnering with thermophilic ANME-1c at 70 °C (ref. ^60^). Thus, Thermodesulfobacteria represent a new group of partner organisms for alkane-oxidizing archaea at high temperatures. In contrast to *Ca*. Alkanophaga, *Ca*. T. syntrophicum encodes and expressed several MHCs, which could support DIET for both partners.

All currently available *Ca*. Alkanophaga sequences originate from the Guaymas Basin, a thoroughly studied hydrothermal vent area hauling heated fluids rich in alkanes^61^. We suspect two main reasons for this apparent absence in other environments. First, until recently, microbial community studies have mostly focused on 16S ribosomal (r)RNA gene amplification and sequencing, a method depending heavily on primer choice^62^. We discovered a mismatch of the commonly used archaeal primer Arch915 (5′-GTGCTCCCCCGCCAATTC**C**T-3′^63^, mismatch in bold) to the 16S rRNA gene sequences of *Ca*. Alkanophaga, which probably produces an artificial underrepresentation of *Ca*. Alkanophaga in public databases. Second, sequencing data from other environments similar to the Guaymas Basin, that is, heated oil reservoirs with sulfate supply, remains scarce. Many of these reservoirs, often buried kilometres deep within the subsurface, are extremely hard to access^64^. In addition, the risk of contamination from the upper biosphere during sampling increases with depth, which might conceal the native community^64^. Still, sampling technologies have greatly improved in recent years, and the focus has shifted from amplification-based 16S rRNA gene to shotgun metagenome studies, which should facilitate a more accurate molecular characterization of reservoir microorganisms. Thus, future studies may disclose the coexistence and activity of *Ca*. Alkanophaga and *Ca*. T. syntrophicum in other heated, petroleum-rich environments.

## Methods

All chemicals were of analytical grade and obtained from Sigma Aldrich, unless otherwise stated. All incubations were done under gentle shaking (40 r.p.m.) in the dark.

### Cultivation of anaerobic thermophilic alkane degraders

The push core used for anoxic cultivations was collected with submersible *Alvin* during RV *Atlantis* cruise AT42-05 in the Guaymas Basin (Gulf of California, Mexico) (dive 4,991, core 15, 27° 00′ 41.1″ N, 111° 24′ 16.3″ W, 2,013 m water depth, 17 November 2018). While shipboard, the push core was transferred to a sealed glass bottle, purged with argon and stored at 4 °C. In the home laboratory, an anoxic sediment slurry was prepared with synthetic sulfate-reducer medium (SRM)^65^, using a ratio of 10% sediment and 90% SRM (v/v), and distributed in 100 ml portions into culture bottles. Cultures were supplemented with 200 μl liquid alkane (C_5_-C_14_) in duplicates. For the C_5_-C_10_ alkanes, 4 ml 2,2,4,4,6,8,8-heptamethylnonane (HMN) were added to mitigate potential toxic effects of the substrate^66^. A substrate-free culture served as a negative control. Headspaces were filled with N_2_:CO_2_ (90:10; 1 atm overpressure) and incubated at 70 °C.

Sulfide production was measured every 2–4 weeks using a copper sulfate assay^67^. Once sulfide concentrations reached 12–15 mM, cultures were diluted 1:3 with SRM and supplied with fresh substrate. Activity doubling times were determined from the development of sulfide concentrations during the first two dilutions. Sulfide concentrations over time were displayed using a logarithmic (base 2) *y* axis. An exponential trend line with the formula $y=n\times{e}^{{mx}}$ was generated. Per definition, the doubling time equals$\,\frac{\mathrm{ln}(2)}{m}$.

### Quantitative substrate turnover experiment

Triplicate 100 ml dilutions with 20 ml headspace were prepared from C_6_- and C_14_-oxidizing cultures, supplied with substrate and incubated at 70 °C, complemented by a substrate-free negative control. Sulfate and DIC concentrations were measured from weekly subsamples until the cultures had reached sulfide concentrations of ≥15 mM. Samples were sterile filtered using a GTTP polycarbonate filter (0.2 μM pore size; Millipore). For DIC measurements, 1 ml filtrate was transferred into synthetic-air-purged 12 ml Exetainer vials (Labco) filled with 100 µl phosphoric acid (45%). After 10 h of equilibration, headspace DIC was measured by isotope ratio infrared spectroscopy (Thermo Fisher; Delta Ray IRIS with URI connect and Cetac ASX-7100 autosampler) with standards of known concentration. To determine sulfate concentrations, 1 ml of the filtrate was fixed in 0.5 ml 100 mM zinc acetate. The sample was centrifuged and the clear supernatant was diluted 1:50 in deionized water. Sulfate was measured by ion chromatography (930 compact IC, Metrohm) against standards with known concentrations.

### DNA extraction and short-read sequencing

DNA was extracted from pellets of 25 ml culture samples collected after the third dilution, using a modified SDS-based extraction method as previously described^68^. Total DNA yield per sample, determined by fluorometric DNA concentration measurement, ranged from 0.9 μg to 3.6 μg. Samples were sequenced at the Max Planck-Genome-Centre (Cologne, Germany). C_6_-C_14_ culture samples were sequenced as 2 × 250 paired-end reads on an Illumina HiSeq2500 sequencing platform. The C_5_ culture sample was sequenced later because of slower growth, together with a sample of the sediment slurry before incubation, by which time the sequencing facility had changed their settings to 2 × 150 bp paired-end reads on an Illumina HiSeq3000 platform. Between 4,140,953 and 4,234,808 raw reads were obtained per culture sample. From the original slurry, 3,130,329 reads were gained.

### Short-read DNA data analysis

Reads from short-read metagenome sequencing were quality-trimmed using BBDuk (included in BBMap v.38.79; https://sourceforge.net/projects/bbmap/; minimum quality value: 20, minimum read length: 50). Reads of the C_6_-C_14_ samples were coassembled using SPAdes (v.3.14.0; https://github.com/ablab/spades)^69^, running BayesHammer error correction and *k*-mer increments (21, 33, 55, 77, 99 and 121) with default settings. The output scaffolds were reformatted using anvi’o (v.7; https://github.com/merenlab/anvio/releases/)^70^, simplifying names and removing contigs shorter than 3,000 bps. Trimmed reads were mapped back to the reformatted scaffolds using Bowtie2 (v.2.3.2; http://bowtie-bio.sourceforge.net/bowtie2/index.shtml)^71^ in the local read alignment setting. Sequence alignment map files were converted to binary alignment map (BAM) files with SAMtools (v.1.5; http://samtools.sourceforge.net/)^72^ and indexed with anvi’o. A contigs database was created from the reformatted scaffolds and profile databases were generated for each sample with anvi’o. Profile databases were merged, enforcing hierarchical clustering. Hidden Markov model (HMM) searches were run via anvi’o on the contigs database to detect genes encoding for Mcrs/Acrs, Wood-Ljungdahl pathway and DSR. Taxonomies for open reading frames were imported into the contigs database using the Centrifuge classifier (v.1.0.2-beta; https://ccb.jhu.edu/software/centrifuge/)^73^. The contigs database was inspected in the anvi’o interactive interface, which clusters the contigs hierarchically on the basis of sequence composition and differential coverage, thereby indicating their relatedness to each other^70^. Binning was performed manually in the interface by clicking branches of the dendrogram in the centre of the interface and using the GC content, mean coverage in the samples, gene taxonomy and real-time statistics on completion and redundancy based on single-copy core genes as guides. The dendrogram branches were followed systematically in a counterclockwise direction to obtain the maximum number of bins. Bin quality was assessed again with CheckM (v.1.1.3; https://ecogenomics.github.io/CheckM/)^74^ and only bins with completeness >50% and redundancy <10% were kept. Taxonomies were assigned to these metagenome-assembled genomes (MAGs) using GTDB-Tk (v.1.5.1; https://github.com/Ecogenomics/GTDBTk)^75^. All manually generated MAGs were refined with anvi’o to minimize contamination. We identified MAGs 1 and 4 as the likely alkane oxidizers and MAG 24 as the likely sulfate reducer based on their mean coverages and HMM hits. To increase the completeness of these three MAGs, an iterative reassembly loop (https://github.com/zehanna/MCA70_analysis/targeted_reassembly_loop.sh) was performed. Therein, the trimmed reads were repeatedly mapped to the refined MAG using BBMap with a minimum alignment identity of 97%. Mapped reads were then assembled using SPAdes. The assembly was quality-checked with CheckM and used as a new reference file to map the trimmed reads to. After performing 25 iterations of this loop, the assembly with the highest quality (that is, highest completeness, lowest contamination and lowest strain heterogeneity) was selected for further analysis. Final MAGs were annotated with Prokka (v.1.14.6; https://github.com/tseemann/prokka)^76^ and the anvi’o-integrated databases NCBI clusters of orthologous genes (COGs)^77^, Kyoto Encyclopedia of Genes and Genomes (KEGG)^78^, Protein Families (Pfams)^79^ and KEGG orthologues HMMs (KOfams)^80^. A bash script (https://github.com/zehanna/MCA70_analysis/CxxCH_scan.sh) was run to search for the haem-binding CxxCH amino acid motif^81^ in the translated gene sequences of the three MAGs. Selected translated gene sequences were exported for gene calls from the contigs database with anvi’o and compared via the BLASTp^82^ web interface (http://www.ncbi.nlm.nih.gov/blast).

Relative abundances of the MAGs were calculated by mapping the trimmed reads to the manually curated and refined MAGs with CoverM (v.0.6.1; https://github.com/wwood/CoverM) in genome mode including the dereplication flag using the default aligner Minimap2 (v.2.21; https://docs.csc.fi/apps/minimap2/) in short-read mode, discarding unmapped reads. The final relative abundance of each MAG is the percentage of the MAG in the mapped fraction of each sample. ANIs between MAGs were calculated with FastANI (v.1.32; https://github.com/ParBLiSS/FastANI).

Because of later sequencing, the original slurry and C_5_ samples were treated separately from the previously sequenced samples and assembled individually. We could not obtain quality MAGs for the original slurry sample; therefore, we estimated the phylogenetic composition on the basis of reconstructed small subunit ribosomal RNAs (SSU rRNAs) mapped against the SILVA SSU reference database (v.138.1)^83^ with phyloFlash (v.3.4.1; https://github.com/HRGV/phyloFlash)^84^. For the C_5_ sample, the same procedure as for the previously sequenced culture samples was followed. The identity (ANI ≥ 95%; ref. ^85^) of the *Ca*. Alkanophaga volatiphilum and *Ca*. Thermodesulfobacterium syntrophicum MAGs from the C_5_ sample, MAG 4_1 and MAG 24_1, respectively, to the previously reconstructed ones was confirmed via FastANI.

To estimate relative abundances of *Ca*. Alkanophaga and *Ca*. T. syntrophicum MAGs in the original slurry, the trimmed reads of the original slurry were mapped to the MAGs with CoverM.

### Construction of phylogenomic trees for archaea and bacteria

The archaeal tree was constructed using 98 publicly available Halobacteriota and Thermoproteota genomes (Supplementary Table 10) from NCBI plus the *Ca*. Alkanophaga MAGs from this study. For the bacterial tree, 121 publicly available Desulfobacterota and Bipolaricaulota genomes (Supplementary Table 10) and the *Thermodesulfobacterium* MAG from this study were included. Trees were based on the concatenated alignment of 76 single-copy core genes (SCG) for archaea and 71 SCGs for bacteria. Alignments were generated with anvi’o, which uses the multiple sequence alignment tool MUSCLE^86^ (v.5.1; https://github.com/rcedgar/muscle). Trees were calculated with RAxML (randomized accelerated maximum likelihood) (v.8.2.12; https://cme.h-its.org/exelixis/web/software/raxml/)^87^ using the PROTGAMMAAUTO model and autoMRE option, which required 50 iterations to reach a convergent tree for both alignments. Trees were visualized with the Interactive Tree of Life online tool (https://itol.embl.de/)^88^. To resolve taxonomic levels, the *Ca*. Alkanophaga MAGs were compared to the ANME-1 and *Ca*. Syntrophoarchaeales MAGs included in the tree by calculating average amino acid identities (AAIs) using the aai_wf feature of the CompareM software (v.0.1.2; https://github.com/dparks1134/CompareM) with default settings.

### In situ hybridization and microscopy

Culture samples were fixed in 1% formaldehyde for 1 h at r.t., washed twice in 1× PBS and stored in 1× PBS-ethanol (1:1 v/v) at −20 °C. Aliquots were filtered onto GTTP polycarbonate filters (0.2 μM pore size; Millipore). Filters were embedded in 0.2% agarose. For permeabilization, three consecutive treatments were performed: (1) lysozyme solution (0.05 M EDTA (pH 8.0), 0.1 M Tris-HCl (pH 7.5) and 10 mg ml^−1^ lysozyme in MilliQ-grade deionized water) for 1 h at 37 °C; (2) proteinase K solution (0.05 M EDTA (pH 8.0), 0.1 M Tris-HCl (pH 7.5) and 7.5 μg ml^−1^ proteinase K in MilliQ) for 10 min at r.t.; and (3) 0.1 M HCl solution for 5 min at r.t. Endogenous peroxidases were inactivated using 0.15% H_2_O_2_ in methanol for 30 min at r.t. A specific probe was designed to exclusively target the *Ca*. Alkanophagales clade. Therefore, the *Ca*. Alkanophaga 16S rRNA gene sequences were added to the SILVA SSU reference database (v.138.1) using the ARB software^89^ (v.7.1; http://www.arb-home.de/home.html). A subtree containing all ANME-1 16S rRNA gene sequences, plus the two sequences from *Ca*. Alkanophaga, was calculated using RAxML (v.8; https://cme.h-its.org/exelixis/web/software/raxml/) with 100 bootstrap replicates, a 50% similarity filter, the GTRGAMMA model and *Methanocella* as outgroup. The probe was generated using the probe design feature with these parameters: length of probe, 19 nucleotides; temperature, 50–100 °C; GC content, 50–100%; *E. coli* position, any; max. non-group hits, 5; min. group hits, 100%. Criteria for candidate probes were: GC content lower than 60%, lowest possible number of matches to non-group species with decreasing temperature, at least one mismatch to non-group species. We ordered a probe that fit these criteria (Aph183) with the sequence 5′-GCATTCCAGCACTCCATGG-3′ from Biomers. For bacteria, the general probe combination EUBI-III (I: 5-GCTGCCTCCCGTAGGAGT-3; II: 5-GCAGCCACCCGTAGGTGT-3; III: 5-GCTGCCACCCGTAGGTGT-3)^90^ was applied. Probe working solution (50 ng µl^−1^) was diluted 1:300 in hybridization buffer containing 30% formamide for Aph183 and 35% formamide for EUBI-III. Probes were hybridized at 46 °C for 3–4 h. Signals were amplified with tyramides labelled with Alexa Fluor 488 for bacteria and Alexa Fluor 594 for *Ca*. Alkanophaga (Thermo Fisher) for 45 min at 46 °C. For double hybridizations, peroxidases from the first hybridization were inactivated using 0.30% H_2_O_2_ in methanol for 30 min at r.t. before the second hybridization and amplification. Filters were analysed via epifluorescence microscopy (Axiophot II imaging; Zeiss). Images were captured with the AxioCamMR camera and the AxioVision software included in the microscope. Images were processed using ImageJ (v.1.49, https://imagej.nih.gov/ij/), where the colour of Alexa488 was changed to cyan to improve accessibility.

### Phylogenetic analysis of proteins involved in alkane oxidation in *Ca.* Alkanophaga

For the *mcrA* tree, the six full-length *mcrA* sequences of *Ca*. Alkanophaga were aligned with 347 publicly available *mcrA* sequences. For the *mer* and the *metF* trees, *Ca*. Alkanophaga sequences were added to publicly available alignments in ref. ^33^ (*mer*: Fig04B; *metF*: Fig05C of Supplement S1). Sequences were aligned with MAFFT (multiple alignment using fast Fourier transform) (v.7.475; https://mafft.cbrc.jp/alignment/software/)^91^. Alignments were trimmed with SeaView (v.5; http://doua.prabi.fr/software/seaview)^92^. For the *mcrA* tree, sequences shorter than 450 amino acids were removed after trimming, after which 337 sequences remained (Supplementary Table 10). Trees were calculated with RAxML (v.8.2.4) using the PROTGAMMAAUTO model, which assigned LG with empirical base frequencies as amino acid model and the autoMRE option for bootstraps, which required 300, 550 and 400 iterations to reach a consensus tree for the *mcrA*, *mer* and *metF* alignments, respectively. Trees were visualized with the Interactive Tree of Life online tool (https://itol.embl.de/)^88^.

### RNA extraction and short-read sequencing

For total RNA extraction, 10 ml of culture material collected after the third dilution at the exponential growth stage were filtered through an RNAse-free cellulose nitrate filter (pore size 0.45 μm; Sartorius). Immediately after filtration, filters were incubated with 5 ml RNAlater for 30 min. RNA was extracted from filters using the Quick-RNA miniprep kit (Zymo Research). DNA was digested without RNase inhibitor. No rRNA depletion step was performed. Between 0.3 and 1.3 μg of total RNA were obtained per sample as determined by fluorometric RNA concentration measurement. Samples were sequenced as 2 × 250 (C_5_: 2 × 150) paired-end reads at the Max Planck-Genome-Centre on the Illumina HiSeq2500 (C_5_: Illumina HiSeq3000) sequencing platform. Between 4,043,349 and 4,785,231 raw reads were obtained per sample.

### Short-read RNA data analysis

Reads from metatranscriptome sequencing were quality-trimmed using BBDuk (included in BBMap v.38.79). Trimmed reads were mapped to the concatenated *Ca*. Alkanophaga MAGs to minimize unspecific mapping because of the high similarity of the two MAGs and to the *Ca*. Thermodesulfobacterium syntrophicum MAG using BBMap (v.38.87) with minimal alignment identity of 98%. Mapped reads were counted using featureCounts (v.1.4.6-p5; http://subread.sourceforge.net/)^93^ with minimum required number of overlapping bases and minimum mapping quality score of 10, counting fragments instead of reads.

Before normalization, rRNA reads were excluded. Fragments were first normalized to gene length, yielding fragments per kilobase (FPK).

2 $${{\rm{FPK}}}_{i}=\,\frac{{C}_{i}}{{L}_{i}\,}\,$$

The centred-log ratio (CLR) was calculated as the base-10 logarithm of read count *C*_*i*_ of gene *i* normalized by gene length *L*_*i*_ in kilobases and divided by the geometric mean of all read counts *C*_1_ − *C*_*n*_ normalized by their respective gene length *L*_1_ − *L*_*n*_.

3 $${{\mathrm{CLR}}}_{i}=\log \left(\frac{\frac{{C}_{i}+0.5}{{L}_{i}}}{\,\sqrt[n]{\frac{({C}_{1}+0.5)}{{L}_{1}}\,\times \,\frac{({C}_{2}+0.5)}{{L}_{2}}\times \ldots \,\times \frac{({C}_{n}+0.5)}{{L}_{n}}}}\right)\,$$

### Test of a selective Mcr inhibitor on culture activity

Duplicates of C_6_- and C_14_-oxidizing culture were supplied with substrate and 5 mM (final concentration) BES. A control culture was supplied with substrate but not with BES. Cultures were incubated at 70 °C and sulfide concentrations were measured until the control cultures had reached >15 mM sulfide.

### Metabolite extraction

Metabolite samples were collected at sulfide levels of 10–14 mM. An 80 ml culture sample of each substrate was pelleted via centrifugation (15 min, 3,100 × *g*, 4 °C). Supernatants were removed, pellets were resuspended in 1 ml of acetonitrile:methanol:water (2:2:1 v/v/v) and transferred to bead-beating tubes. Samples were agitated for 15 min on a rotor with vortex adapter at maximum speed. Samples were centrifuged for 20 min at 10,000 × *g* at 4 °C. Clear supernatants were stored at 4 °C.

### Synthesis of authentic alkyl-CoM standards

Coenzyme M (sodium 2-mercaptoethanesulfonate) (0.1 g) was dissolved in 2 ml 25% (v:v) ammonium hydroxide solution and twice the molar amount of bromoalkane was added. We acquired 2- and 3-bromohexane from Tokyo Chemical, and 2-bromotetradecane from Alfa Aesar. Vials were incubated for 6 h at r.t. under gentle shaking on a rotor with vortex adapter. The clear upper phase (1 ml) was collected and stored at 4 °C.

### Mass spectrometry of culture extracts and standards

Culture extracts and standards were analysed using high-resolution accurate-mass mass spectrometry on a Bruker maXis plus quadrupole time-of-flight (QTOF) mass spectrometer (Bruker) connected to a Thermo Dionex Ultimate 3000RS UHPLC system (Thermo Fisher) via an electrospray ionization (ESI) ion source. Sample aliquots were evaporated under a nitrogen stream and re-dissolved in a methanol:water (1:1 v/v) mixture before injection. A 10 μl aliquot of the metabolites was injected and separated on an Acclaim C30 reversed phase column (Thermo Fisher; 3.0 × 250 mm, 3 µm particle size) set to 40 °C using a flow rate of 0.3 ml min^−1^ and the following gradient of eluent A (acetonitrile:water:formic acid, 5:95:0.1 v/v/v) and eluent B (2-propanol:acetonitrile:formic acid, 90:10:0.1 v/v/v): 0% B at 0 min, then ramp to 100% B at 30 min, hold at 100% B until 50 min, followed by re-equilibration at 0% B from 51 min to the end of the analysis at 60 min to prepare the column for the next analysis. The ESI source was set to the following parameters: capillary voltage 4,500 V, end plate offset 500 V, nebulizer pressure 0.8 bar, dry gas flow 4 l min^−1^, dry gas heater 200 °C. The QTOF was set to acquire full scan spectra in a mass range of *m*/*z* 50–600 in negative ionization mode. The C_14_ culture extract was additionally analysed in tandem mass spectrometry mode, and mass spectra of the fragmentation products of *m*/*z* 337.1877 isolated in a window of 3 Da and fragmented with 35 eV were acquired. Every analysis was mass-calibrated to reach mass accuracy of 1–3 ppm by loop injection of a calibration solution containing sodium formate cluster ions at the end of the analysis during the equilibration phase and using the high-precision calibration algorithm. Data were processed using the Compass DataAnalysis software package v.5.0 (Bruker).

### Substrate range tests

Cultures originally grown with C_6_ and C_14_ were diluted 1:10 in fresh SRM. Dilutions were supplemented with alkanes between C_5_ and C_14_ for which growth had not been confirmed yet, and with shorter (C_3_ and C_4_) and longer (C_16_-C_20_) alkanes (Table 1).

**Table 1:** Overview over substrate range test with *Ca.* Alkanophaga cultures

Organism	Originally consumed C_x_-*n*-alkanes	Culture used as inoculum	Tested C_x_-*n*-alkanes
*Ca.* Alkanophaga volatiphilum	C_5_, C_6_, C_7_	C_6_	C_3_, C_4_, C_8_, C_9_, C_10_, C_11_, C_12_, C_13_, C_14_, C_15_, C_16_, C_18_, C_20_
*Ca.* Alkanophaga liquidiphilum	C_8_, C_9_, C_10_, C_12_, C_14_	C_14_	C_3_, C_4_, C_11_, C_13_, C_15_, C_16_, C_18_, C_20_

A negative (inoculated culture without substrate) and a positive (inoculated culture supplied with substrate with which the culture was originally grown) control were also set up. Cultures were incubated at 70 °C and activity was tracked via sulfide measurements. Once sulfide concentrations reached >10 mM, cultures were diluted 1:3 with SRM. The procedure was repeated and incubations that showed sustained activity over two dilutions were considered successful.

### Hydrogen production measurements

C_6_ and C_14_ cultures were divided into two 20 ml aliquots in 156 ml serum bottles. One aliquot was left untreated, the other one was treated with 10 mM (final concentration) sodium molybdate. Hydrogen was measured by injecting 1 ml of headspace sample into a Peak Performer 1 gas chromatograph (Peak Laboratories). Measurements were taken in 1 h intervals up to 8 h after the start of the experiment. A final measurement round was conducted from 24 h to 30 h in 2 h intervals.

### Test of the effect of addition of hydrogen and formate on culture activity

Two replicates of C_6-_ and C_14_-oxidizing cultures were supplied with substrate and with 10% H_2_ in the headspace or 10 mM (final concentration) sodium formate in the medium. A control culture was supplied only with substrate. Cultures were incubated at 70 °C and sulfide concentrations were measured until the control cultures had reached ≥15 mM sulfide.

### Transmission electron microscopy

C_6_ and C_14_ culture (100 ml) were collected at 1,000 × *g* using a Stat Spin Microprep 2 table top centrifuge. Cells were transferred to aluminium platelets (150 µm depth) containing 1-hexadecene^94^. Platelets were frozen using a Leica EM HPM100 high-pressure freezer (Leica). Frozen samples were transferred to a Leica EM AFS2 automatic freeze substitution unit and substituted at −90 °C in a solution containing anhydrous acetone and 0.1% tannic acid for 24 h, and in anhydrous acetone, 2% OsO_4_ and 0.5% anhydrous glutaraldehyde (Electron Microscopical Science) for a further 8 h. After further incubation over 20 h at −20 °C, samples were warmed to +4 °C and subsequently washed with anhydrous acetone. Samples were embedded at room temperature in Agar 100 (Epon 812 equivalent) at 60 °C for 24 h. Thin sections (80 nm) were counterstained using Reynolds lead citrate solution for 7 s and examined using a Talos L120C microscope (Thermo Fisher).

### Temperature range tests

Aliquots of C_6_- and C_14_-oxidizing cultures were diluted 1:6, supplied with substrate and incubated at 60–90 °C in 5 °C increments. Sulfide production was tracked until the 70 °C cultures had reached >10 mM sulfide.

### Availability of biological materials

Official culture collections do not accept syntrophic enrichment cultures, but G.W. will maintain the cultures. Non-profit organizations can obtain samples upon request.

### Reporting summary

Further information on research design is available in the Nature Portfolio Reporting Summary linked to this article.

## Supplementary information

Reporting Summary

Peer Review File

Supplementary Tables 1–10 Additional information on culture physiology and results from metagenome analyses.

## Data Availability

The following databases were used in this study: SILVA SSU reference database (v.138.1; https://www.arb-silva.de/documentation/release-1381/), NCBI COGs (https://www.ncbi.nlm.nih.gov/research/cog-project/), KEGG (https://www.genome.jp/kegg/kegg1.html), Pfam (https://www.ebi.ac.uk/interpro/), KOfam (https://www.genome.jp/tools/kofamkoala/) plus alignments in ref. ^33^ (*mer*: Fig04B; *metF*: Fig05C of Supplement S1; 10.1371/journal.pbio.3001508.s017). MAGs of *Ca*. Alkanophaga (*Ca*. A. volatiphilum: BioSample SAMN29995624, genome accession: JAPHEE000000000; *Ca*. A. liquidiphilum: SAMN29995625, JAPHEF000000000) and *Ca*. Thermodesulfobacterium syntrophicum (SAMN29995626, JAPHEG000000000), the raw reads from short-read metagenome and transcriptome sequencing, the coassembly of the C_6_-C_14_ samples, and the single assemblies of the original slurry and the C_5_ samples (SAMN30593190, Sequence Read Archive (SRA) accessions SRR22214785-SRR22214804) are accessible under BioProject PRJNA862876. The mass spectrometry runs for the detection of alkyl-CoMs have been deposited to the EMBL-EBI MetaboLights database^95^ with the identifier MTBLS7727. Source data are provided with this paper.

## Source data

Source Data Fig. 1 Statistical source data.
Source Data Fig. 2 Statistical source data (Fig. 2a) and alignment of single-copy core genes used for archaeal phylogenomic tree calculation (Fig. 2b).
Source Data Fig. 3 Alignment of *mcrA* sequences used for phylogenetic tree calculation (Fig. 3a) and statistical source data (Fig. 3b).
Source Data Fig. 5 Statistical source data.
Source Data Extended Data Fig. 1 Statistical source data.
Source Data Extended Data Fig. 2 Statistical source data.
Source Data Extended Data Fig. 3 Statistical source data.
Source Data Extended Data Fig. 4 Statistical source data.
Source Data Extended Data Fig. 6 Statistical source data.
Source Data Extended Data Fig. 7 Statistical source data.
Source Data Extended Data Fig. 8 Alignments of *mer* (Extended Data Fig. 8a mer) and *metF* (Extended Data Fig. 8b metF) sequences used for phylogenetic tree calculation.
Source Data Extended Data Fig. 9 Alignment of single-copy core genes used for bacterial phylogenomic tree calculation.

## Code availability

The workflow for metagenome and transcriptome analysis, and the scripts for targeted reassembly and for the search of CxxCH motifs are available under https://github.com/zehanna/MCA70_analysis. Further inquiries about bioinformatic analyses may be directed to the corresponding authors.
